# Horizontally Acquired Genes Are Often Shared between Closely Related Bacterial Species

**DOI:** 10.3389/fmicb.2017.01536

**Published:** 2017-08-25

**Authors:** Evgeni Bolotin, Ruth Hershberg

**Affiliations:** Rachel and Menachem Mendelovitch Evolutionary Processes of Mutation and Natural Selection Research Laboratory, The Rappaport Family Institute for Research in the Medical Sciences, Department of Genetics and Developmental Biology, Technion-Israel Institute of Technology Haifa, Israel

**Keywords:** gene content, pangenome, bacterial evolution, horizontal gene transfer, genome composition

## Abstract

Horizontal gene transfer (HGT) serves as an important source of innovation for bacterial species. We used a pangenome-based approach to identify genes that were horizontally acquired by four closely related bacterial species, belonging to the *Enterobacteriaceae* family. This enabled us to examine the extent to which such closely related species tend to share horizontally acquired genes. We find that a high percent of horizontally acquired genes are shared among these closely related species. Furthermore, we demonstrate that the extent of sharing of horizontally acquired genes among these four closely related species is predictive of the extent to which these genes will be found in additional bacterial species. Finally, we show that acquired genes shared by more species tend to be better optimized for expression within the genomes of their new hosts. Combined, our results demonstrate the existence of a large pool of frequently horizontally acquired genes that have distinct characteristics from horizontally acquired genes that are less frequently shared between species.

## Introduction

Gene gain is a major driving force in the evolution of bacterial genomes (Ochman et al., 2000; Gogarten et al., 2002; Thomas and Nielsen, 2005; Boto, 2010; Vogan and Higgs, 2011; Arber, 2014). In addition to gene duplication and the de-novo creation of novel genes, the major contributor to bacterial gene gain is horizontal gene transfer (HGT). The molecular mechanisms of HGT were discovered many decades ago through the pioneering studies of Oswald Avery, Joshua Lederberg, Edward Tatum, Norton Zinder and others (Lederberg and Tatum, 1946; Zinder and Lederberg, 1952; Boto, 2010; Arber, 2014; Cobb, 2014). Subsequent advances in genome sequencing technologies enabled comparative analysis of numerous complete bacterial genomes, showing that HGT is a frequent and ubiquitous phenomenon among prokaryotes (Lawrence, 1999; Eisen, 2000; Ochman et al., 2000; Boto, 2010).

The fate of newly horizontally acquired genes depends on their impact on the host genome. Acquired genes that do not contribute positively to the fitness of their new host will often be purged from the population by natural selection and / or by genetic drift (Kurland et al., 2003; van Passel et al., 2008; Kuo and Ochman, 2009; Nielsen et al., 2014). On the other hand, acquired genes with beneficial effects on the host (e.g., antibiotic resistance and virulence genes) are more likely to persist and increase in frequency within a bacterial population (Nielsen et al., 2014; Burmeister, 2015; von Wintersdorff et al., 2016). One factor that may affect the ability of horizontally acquired genes to be maintained within their new host genome is their codon usage (Angov, 2011; Plotkin and Kudla, 2011; Tuller et al., 2011; Park and Zhang, 2012). The genetic code is redundant, meaning that many amino acids are encoded by more than one codon. Different codons encoding the same amino acid are referred to as synonymous codons (Angov, 2011; Plotkin and Kudla, 2011). Within a given genome certain synonymous codons will be used more frequently than others (a phenomenon known as “codon usage bias”). This is due both to the background substitution biases of a genome (which can lead to differences in the usage of codons, depending on their nucleotide composition) (Hershberg and Petrov, 2009) and to natural selection favoring the usage of certain synonymous codons over others (Hershberg and Petrov, 2008; Angov, 2011; Plotkin and Kudla, 2011; Hershberg, 2016). Natural selection affects codon usage because different tRNAs vary in their concentrations leading to variation in the efficiency and accuracy with which the codons they recognize are translated (Hershberg and Petrov, 2008; Hershberg, 2016). Horizontally acquired genes that use codons that better match the codon usage of their new host genome will be better optimized for expression within the context of that genome and will confer less of a fitness cost on their new host (Angov, 2011; Plotkin and Kudla, 2011; Tuller et al., 2011; Park and Zhang, 2012). Such horizontally acquired genes should therefore have higher chances of being maintained.

A useful tool in studying the evolution of bacterial gene content is the pangenome (Tettelin et al., 2005; Lapierre and Gogarten, 2009; Rouli et al., 2015; Vernikos et al., 2015). The pangenome represents the collection of all genes found within the genomes of an investigated bacterial lineage (Tettelin et al., 2005, 2008; Vernikos et al., 2015). The pangenome of a specific lineage (usually a species) is constructed by clustering orthologous genes from the investigated genomes into groups called “pangenes.” Once all the orthologs are clustered, it is possible to draw a pangenome plot depicting the distribution of pangene frequencies. Previous studies have shown that the typical pangenome plot of a bacterial species is asymmetrically U-shaped, with high frequencies of pangenes found at either few or most of the species' strains and low frequencies of pangenes found in an intermediate number of strains (Lapierre and Gogarten, 2009; Collins and Higgs, 2012; Lobkovsky et al., 2013; Bolotin and Hershberg, 2015). Such a distribution of pangene frequencies suggests that pangenes found only in a few strains (from here on referred to as “rare” pangenes or “rares”) are likely to represent genes that were introduced into the species by HGT (Bolotin and Hershberg, 2015). After all, explaining the presence of “rare” pangenes by loss of vertically inherited genes would require a high number of gene loss events, while gene gain needs to happen only once or a few times to explain the existence of such rare pangenes. Also, if we try to explain the presence of “rare” pangenes primarily by gene loss, it is unclear why pangenes found at intermediate frequencies would be so rare. For there to be many rare pangenes, but few intermediate pangenes due to gene loss, gene loss would often have to occur independently in many strains within a species, but very rarely occur in an intermediate number of strains. Such a pattern of gene loss seems to be very unlikely. Further supporting the notion that “rare” pangenes represent events of gene gain via HGT, we have previously shown that in the pangenomes of clonal bacterial species that undergo little to no HGT, “rare” pangenes are virtually non-existent (Bolotin and Hershberg, 2015). Based on similar reasoning, pangenes found in the majority, but not all, strains of a species are likely to represent vertically inherited, “core” pangenes that have experienced gene loss within some strains of a species, rather than horizontally acquired genes that spread to many strains (Bolotin and Hershberg, 2015). From here on we will refer to genes found in all or in the majority of strains of a species as “extended core” pangenes.

Here, we utilize a pangenomic approach to examine the extent to which horizontally acquired genes tend to be shared between closely related bacterial species. By examining and comparing the pangenomes of four closely related *Enterobacteriaceae* species, we demonstrate that a high proportion of genes acquired by any given species are shared by other species as well. We further demonstrate that acquired genes shared by more species tend to have properties that distinguish them from less frequently shared acquired genes.

## Materials and methods

### Datasets

All genomic data used in this study were downloaded from the NCBI (National Center for Biotechnology Information) database (January 2016) (O'Leary et al., 2016). The four selected species used for analyses are all members of the *Enterobacteriaceae* family that have relatively large numbers of strains sequenced (Table 1). Strains, whose genomes were not fully sequenced or strains that were artificially manipulated and duplicate genomes of the strains that were sequenced more than once were removed from further analyses. *Escherichia coli* and *Shigella* spp. strains were combined into one dataset, since studies indicate that *Shigella* strains are members of the *E. coli* species (Pupo et al., 2000; van den Beld and Reubsaet, 2012; Gordienko et al., 2013). For simplicity sake, in the text the combined *E.coli*- *Shigella* spp. group was referred to simply as “*E. coli.”* The list of all species and strains analyzed is provided in Table S1. A list of strains removed from consideration is provided in Table S2.

**Table 1:** Summary of the pangenome data in the analyzed species.

**Species**	**Number of strains**	**Total number of the pangenes**	**“Rare” pangenes**	**Percent of the “rares” in the pangenome**
*Enterobacter cloacae*	16	9586	5278	55.06
*Escherichia coli*	85	11741	7596	64.70
*Klebsiella pneumoniae*	34	8652	3692	42.67
*Salmonella enterica*	72	9091	4893	53.82

The relatedness of the analyzed species was estimated using the average amino acid identity (AAI) metric (Table S3). For a pair of organisms, AAI measures levels of similarity between sequences of their orthologous proteins, providing a robust measure of genetic relatedness between groups of organisms (Konstantinidis and Tiedje, 2005a,b; Chan et al., 2012). To calculate AAI, representative protein sequences of each pangene within the species' pangenomes (see below) were compared in a pairwise manner using FASTA (Pearson and Lipman, 1988). Orthologous protein pairs were identified then by requiring bi-directional best hit (BBH). The percent of amino acid identity was calculated for each pair of orthologous proteins, and, based on the AAI values for all orthologs in each pair of pangenomes, AAI values for all pairs of pangenomes were calculated. Following the thresholds set by POGO-DB (Lan et al., 2014), only pairs of putative orthologs sharing at least 30% of sequence identity over 70% of protein length and having no more than 1/3 length difference between the lengths of the short and the long protein in the pair were used for AAI calculations.

### Pangenome construction

The pangenomes of the investigated bacterial species were constructed as described in Bolotin and Hershberg (2015) and Bolotin and Hershberg (2016). First, all paralogs and paralog-related genes were removed from all genomes used for pangenome generation. This was done since there are no clear rules how to treat paralogs during pangenome construction and their inclusion may generate computational biases. To identify paralogs, the genome of each strain was compared to itself using FASTA (Pearson and Lipman, 1988), and each gene mapping to any gene other than itself above a defined threshold was considered as a paralog and removed from further analyses, together with the genes it was mapped to. The threshold for considering two genes as paralogs was defined at 80% normalized identity (NI), where NI is defined as:

NI=I*AL/QL

where: I–sequence similarity across the aligned region (%), AL–length of the aligned region and, QL–length of the query sequence.

Next, the genome of each strain was compared to paralog sequences removed from all other strains to identify and remove paralog-related genes—genes that have no paralogs within a given strain, but match paralog sequences from other strains above a threshold of 80% NI. Due to the removal of the paralog genes, paralog-related genes would be under-represented in the pangenome, and their inclusion in the pangenome construction and subsequent analyses may generate artifacts. To ensure that no paralog genes are missed, paralog and paralog-related gene identification was done both at the protein and nucleotide levels. Only genes that were identified as non-paralog and non-paralog-related at both the protein and DNA levels were used for pangenome construction.

Following the paralog removal step, the levels of gene content dissimilarity between the strains of each species were assessed to identify identical or nearly identical strains within each species. Inclusion of many identical, or nearly identical strains in the pangenome of a species may cause certain pangenes to appear at higher frequencies even though they represent acquired genes, thus limiting our ability to detect them. To avoid this bias, genomes of strains having low levels of gene content diversity were merged together and treated as a single strain for the purposes of pagenome construction. The levels of gene content similarity were assessed using the genomic fluidity metric, as described in (Bolotin and Hershberg, 2015). Briefly, for a pair of strains genomic fluidity represents the ratio between the number of genes unique to each strain and the total number of genes in both strains. Genomes of strains having genomic fluidity ≤1% were considered identical or nearly identical and merged. RefSeq IDs of strains that were merged together are listed in Table S4.

Merging of clusters of nearly identical strains within each species was done similarly to pangenome construction. Within each cluster of identical or nearly identical strains, a randomly selected genome served as an initial library. All other strains were compared to the initial library in a pairwise manner using FASTA (Pearson and Lipman, 1988). During the comparison genes identified as orthologs were combined into a single cluster, while sequences having no matches in the library were added to it, so the next strain was compared against the expanded library. The first sequence within each gene cluster to which all subsequent sequences were added was taken as the representative of the cluster. The resulting library of gene clusters across nearly identical strains was considered as a single “strain” for pangenome construction and subsequent analyses.

To initiate the construction of a pangenome for a certain species, we selected at random the genome of one of the strains of the species to serve as the initial library. All remaining strains were then compared iteratively to the library using FASTA (Pearson and Lipman, 1988) in a pairwise manner. During each pairwise comparison identified orthologs were combined into pangene groups. The first sequence in the pangene group, to which all other sequences were added during iterative comparisons, was taken as a representative sequence of the pangene. The query sequences that didn't find a match in the initial library were added to it, so the comparison of the following genome was done against an expanded library. After all the genomes were compared, the resulting pangenome was compared to itself to minimize computational bias stemming from random choice of the initial library. The generated pangenome was also corrected for additional possible artifacts as in Bolotin and Hershberg (2015) and Bolotin and Hershberg (2016).

### Identification of the shared “rare” pangenes

To examine whether “rare” pangenes from one species were shared by the other studied species, a representative sequence of each “rare” pangene in the species' pangenome was compared to the genomic data (pangenome and paralog sequences removed prior to the pangenome construction, see above) of the three other studied species in a pairwise manner using FASTA (Pearson and Lipman, 1988). A “rare” pangene was considered as shared by a second species, if three conditions were fulfilled:

1. It found a match in the second species with a percent identity above a set threshold [thresholds were set to 55, 65, or 75% depending on the stage of the analysis (see results section)],
2. The difference in the lengths of the query protein and its match was not above 1/3,
3. The length of the aligned region between the query and its match was at least 70% of the length of the shortest between them.

### Generation of “rare” pangene sharing saturation curves

**Figure 1:**
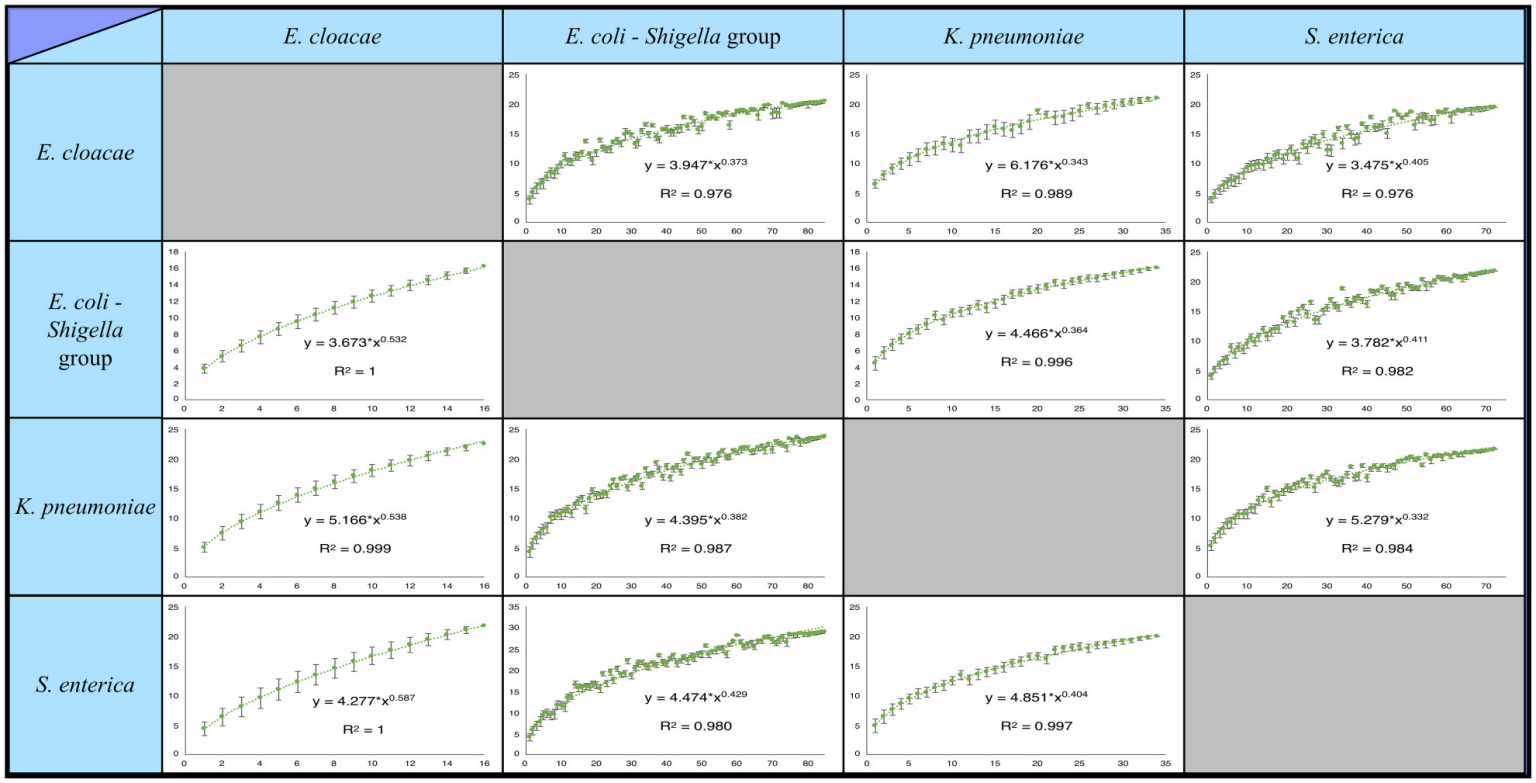
Percentage of rare pangenes shared between the studied *Enterobacteriaceae* species is likely underestimated. Presented are saturation plots for each possible species pair combination. The average percent of “rare” pangenes, out of all rare pangenes of one species (Y-axis) found in a second species is depicted as a function of the number of second species strains that were included in the analysis (X-axis). Error bars represent deviation of the number of the shared “rares” in the individual combinations of strains from the average (see Materials and methods). The resulting data was fitted using Heap's law. For all species pairs, inclusion of more second species strains into the analysis continues to unravel additional shared “rare” pangnes with no evident saturation.

The pangenomes of each pair of species were compared, as described above, and “rare” pangenes of species A shared by pangenome of species B were identified. For each matching pangene in species B we checked in which strains it is found. This allowed us to calculate how many “rare” pangenes of species A can be found in the second species if we take into account only a subset of strains from species B. For each number of strains in species B, from 1 to N (N being all strains), the average number of “rare” pangenes from species A shared by species B was calculated for all possible combinations of the strains. If for a certain number of strains, the number of possible combinations was over 1,000,000, the average number of shared “rare” was calculated over a randomly selected set of 1,000,000 combinations. The resulting curves (Figure 1) were fitted using Heap's law in the form:

$$SP(\%)=K*n^{\beta },$$

Where: SP–percent of **s**hared “rare” **p**angenes; n–**n**umber of strains in species B; K and β–free parameters determined by the fit.

### Evolutionary conservation analysis

For the conservation analysis of the “rare” pangenes beyond the four analyzed *Enterobacteriaceae* species, all completely sequenced and annotated bacterial genomes were downloaded from the NCBI database (January 2016) (O'Leary et al., 2016). Representative protein sequences of each “rare” pangene within a species' pangenome were compared to the protein sequence data of each bacterium in the downloaded database using FASTA (Pearson and Lipman, 1988). If the query protein sequence identified a hit within a target bacterium above the threshold of 55% sequence identity and alignment length to query protein length ratio was over 2/3, the pangene was considered as present in the target organism. The NCBI taxonomy database (Ncbi Resource Coordinators, 2016) was used to identify the species-level classification of each strain present in the complete bacterial database, allowing us to calculate for each “rare” pangene the number of additional bacterial species in which it was found. If a certain pangene was not found in any additional species outside the four analyzed species, it was marked as conserved by “0” additional species. Statistical significance of differences in the evolutionary conservation values across different sharing groups of “rare” pangenes was calculated using a one-sided non-paired Mann-Whitney test as implemented by the wilcox.test function in R (R Core Team, 2015).

### Codon usage analyses

To calculate ENC' for each “rare” pangene we used the ENCprime program (Novembre, 2002) with default settings. The ENC' value was calculated for each individual gene within a pangene, and then the average ENC' value per pangene was calculated. If for a certain pangene the standard deviation of ENC' values between individual genes was higher than 10%, the pangene was excluded from further analysis. In addition, only data from pangenes in which all genes were at least 100 codons long were included in the analysis.

Optimal codon frequency (FoP) for each “rare” pangene was calculated using a custom Perl script. Similarly to ENC' prime calculation, FoP values per pangene were calculated as an average across FoP values of individual genes belonging to that pangene. A standard deviation of 10% or less between FoP values of individual genes was required to include the pangene in further analysis.

Optimal codons of each species are listed in Table S5. The identity of the optimal codons in *E. coli, K. pneumoniae* and *S. enterica* was taken from the work of Hershberg and Petrov (2009). The identity of the optimal codons in *E. cloacae* was calculated as described in Hershberg and Petrov (2009). Briefly, for each codon family (a codon family being the set of codons encoding the same amino acid), we identified those codons whose frequency correlates significantly and most strongly with the overall levels of codon usage of genes. Codons that are used more frequently as genes become more biased in their codon usage overall are likely to be favored. Spearman correlations between our measure of overall levels of codon bias (ENC') and the relative frequency of each codon within a codon family were calculated separately for each strain of *E. cloacae*. If for different strains different codons within a certain family showed the significant best correlations, all were considered as optimal codons. To avoid inaccuracies in the computation of ENC', protein-coding genes having less than 50 codons were excluded from the calculation. Protein-coding genes in which a certain codon family was represented by less than 10 codons were excluded from the Spearman's test for that codon family.

Statistical significance of differences in the ENC' and FoP values across different sharing groups of “rare” pangenes was calculated using a one-sided non-paired Mann-Whitney test as implemented by the wilcox.test function in R (R Core Team, 2015).

### Analysis of gene length

To analyze differences in gene length among “rare” pangenes belonging to various sharing groups, the length of each pangene was calculated as an average of gene lengths of all genes belonging to that pangene. Next, a non-paired Mann-Whitney test as implemented by the wilcox.test function in R (R Core Team, 2015) was used to calculate the statistical significance of differences observed across different sharing groups of “rare” pangenes

## Results

### Identification of the predicted horizontally acquired “rare” pangenes

In this work, we focused on four species belonging to the *Enterobacteriaceae* family: *Enterobacter cloacae, Escherichia coli, Klebsiella pneumoniae* and *Salmonella enterica*. These species were chosen because of their close phylogenetic relatedness to each other (78–82% average amino acid identity (AAI) between orthologous proteins from each species, see Table S3) and because they each have a high number of fully sequenced strains available for pangenomic analysis (Table 1, Table S1). Based on the genomic data and gene annotations provided by the NCBI we constructed the pangenome of each species (see Materials and Methods). As already discussed in the introduction, pangenes found only in a few strains of a species were likely introduced via HGT. We therefore classified pangenes found in 25% or less of all strains in each species as “rare” pangenes that were probably acquired via HGT. “rare” pangenes represent significant portions of the investigated species' pangenomes (~43–~65% of the pangenes of each species, Table 1), indicating that the analyzed species undergo substantial HGT.

### A high percentage of likely horizontally acquired pangenes are shared among closely related species

To estimate how many “rare” pangenes are unique to each studied species and how many are shared by these species, we compared “rare” pangenes of each species to the genomic content of each of the three other species using FASTA (Pearson and Lipman, 1988), in a pairwise manner. Pangenes that found a match with at least 55% identity to a protein in different species were considered as shared by both species (see Materials and Methods). We found that for the four analyzed species, between 38 and 51% of rare pangenes can be found in at least one additional closely related species (Table 2). Shared “rare” pangenes of one species are most frequently shared by only one additional species, and less frequently—by two or all three other species (Table 2).

**Table 2:** Distribution of the shared “rare” pangenes across the four studied species.

**Species**	**“Rare” pangenes**	**Non-shared “rares” (%)**	**Total shared “rares” (%)**	**Shared with 1 species (%)**	**Shared with 2 species (%)**	**Shared with 3 species (%)**
*Enterobacter cloacae*	5278	58.34	41.66	18.76	12.56	10.34
*Escherichia coli*	7596	62.40	37.60	19.73	10.73	7.14
*Klebsiella pneumoniae*	3692	53.17	46.83	20.88	14.82	11.13
*Salmonella enterica*	4893	49.15	50.85	24.22	15.57	11.06

It was previously found that for many bacteria, including some of the studied species, the pangenome is not closed, meaning that with the sequencing of additional strains new pangenes will be added to the pangenome (Rasko et al., 2008; Gordienko et al., 2013; Holt et al., 2015; Rouli et al., 2015; Maiden and Harrison, 2016; Roer et al., 2016). Thus, despite the high number of strains analyzed in each species, it is still possible that we do not capture the whole diversity of the “rare” pangenome in these species and underestimate the amount of shared “rare” pangenes. To quantify the extent to which we may be missing shared “rare” pangenes, we generated pangene-sharing saturation plots for each pair of species (Figure 1). For a pair of species, A and B, the saturation plot depicts the percent of “rare” pangenes from species A that can be found in species B as a function of the number of analyzed strains in species B (see Materials and Methods and Figure 1). The resulting saturation plots do not show any signs of saturation for the percent of shared “rare” pangenes, even once all available genomes of species B are analyzed. This strongly suggests that our estimations of the shared “rare” pangene content represent only a lower bound, and sequencing additional strains within each species may reveal that a substantial number of additional “rare” pangenes are shared between species.

Further, we examined how the estimated percentage of shared “rare” pangenes in each species changes when we use more stringent identity cut-offs (65 and 75%) to classify pangenes as shared. Under a threshold of 65% identity we found that, for the four studied species, between 33 and 44% of genes were shared in at least one more species (Table S6). Under a threshold of 75% the percentage of shared genes decreased further to between 24 and 35% (Table S6). These results indicate high variability in the degree of relatedness between the shared “rares.” The majority of shared pangenes tend to be highly similar (>75%, which is similar to the overall level of similarity between the studied species) (Table S6), indicating that they were probably acquired from phylogenetically closely related sources. At the same time, some of the shared “rare” pangenes display lower levels of sequence similarity and may represent more distant homologs acquired from more distantly related sources.

The majority of “rare” pangenes of one species found in a different species belong to the “rare” category of pangenes within the second species as well (between 66.4 and 85.5%, for the four considered species, Table 3). This indicates that most “rare” pangenes shared by two species were horizontally acquired by both species. At the same time, “extended core” pangenes and pangenes found at intermediate frequencies (and their homologs) appear to have a much lower propensity of being horizontally acquired by other species.

**Table 3:** Distribution of the shared “rare” pangenes in each species by a pangene category of their homolog in the second species.

**Organism**	***E. cloacae***			***E. coli***			***K. pneumoniae***			***S. enterica***		
	**Rare**	**Middle**	**Ext. core**	**Rare**	**Middle**	**Ext. core**	**Rare**	**Middle**	**Ext. core**	**Rare**	**Middle**	**Ext. core**
*E. cloacae*				81.34	5.97	12.69	67.27	9.79	22.94	81.43	3.91	14.66
*E. coli*	78.94	8.41	12.65				67.93	13.19	18.88	82.02	5.53	12.45
*K. pneumoniae*	79.78	11.26	8.96	81.33	7.33	11.34				76.64	4.29	19.07
*S. enterica*	83.03	7.07	9.90	85.50	5.94	8.56	71.97	13.45	14.58			

### Shared horizontally acquired pangenes are conserved in a larger number of additional bacterial species compared to horizontally acquired pangenes unique to a single species

**Figure 2:**
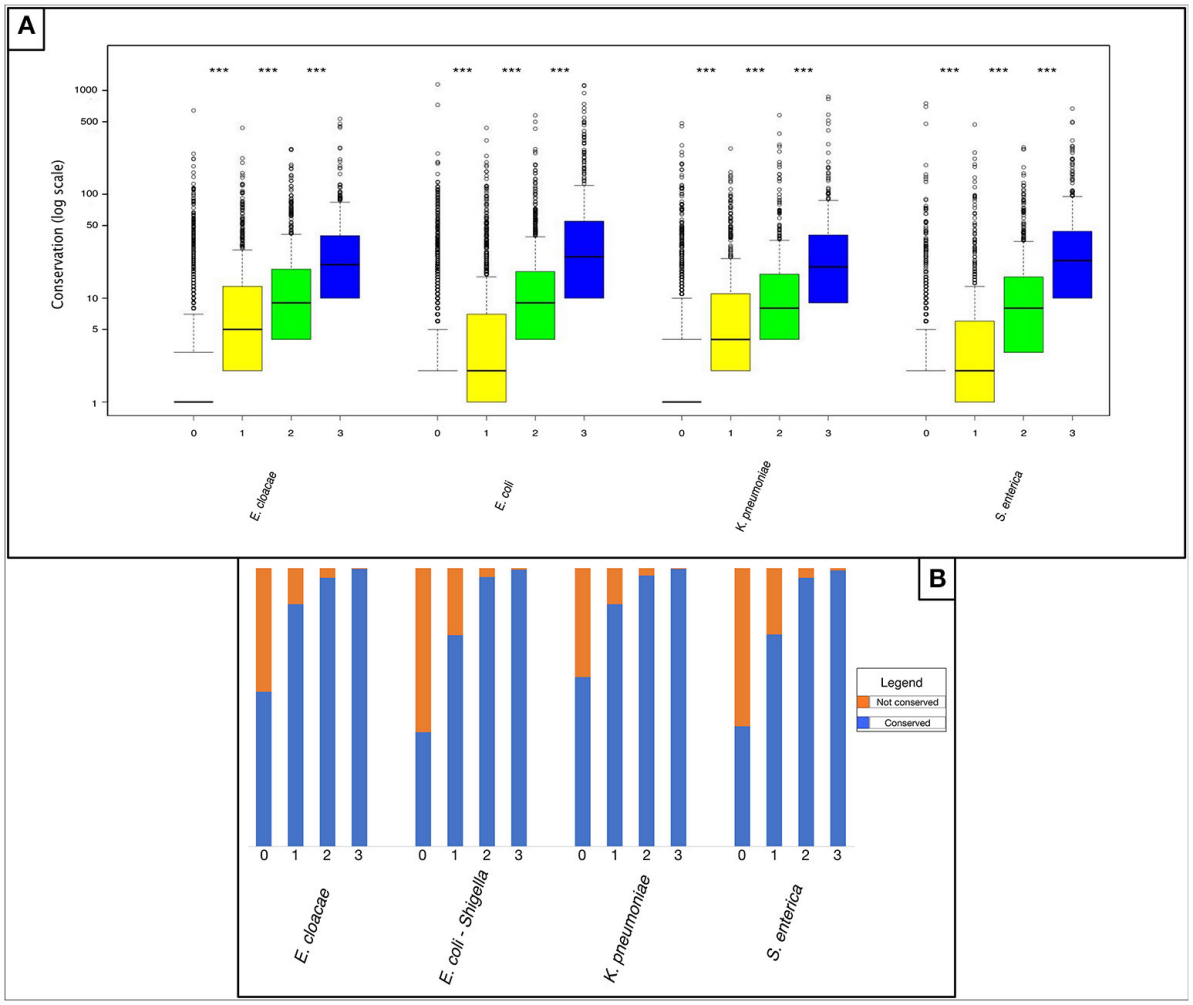
Horizontally acquired genes shared by more of the four studied *Enterobacteriaceae* family species tend to be more conserved in additional species as well. **(A)** Box plot representing the number of species in which “rare” pangenes were found, outside of the four *Enterobacteriaceae* family studied species. For each species four boxes are given: red – “rare” pangenes that are not shared in any of the three other species; yellow—“rare” pangenes shared by one additional species; green—“rare” pangenes shared by two additional species; blue—“rare” pangenes shared by all species. Whisker length for each boxplot represents 1.5 IQR. Statistical significance of differences between the gene-loss groups according to a non-paired, one-sided Mann-Whitney-Wilcoxon test is denoted by: ^***^P ≤ 0.001. The Y-axis is presented in logarithmic scale. **(B)** Bar plot depicting the percent of “rare” pangenes in each sharing group that are not conserved outside the four analyzed species (orange), or that are shared outside the four analyzed species (blue).

We compared non-shared and shared “rare” pangenes with regards to their conservation outside of the four analyzed species. We conducted FASTA (Pearson and Lipman, 1988) comparisons of each pangene against all fully sequenced bacterial genomes downloaded from the NCBI database (O'Leary et al., 2016) and calculated the number of additional bacterial species in which each pangene was present (Materials and Methods). Our results indicate that “rare” pangenes shared by a higher number of analyzed closely related species are also found in a higher number of additional bacterial species, suggesting higher degree of global conservation (*P* < 2.2E-16 according to a non-paired, one-sided Mann-Whitney test for comparisons of all sharing groups, Figure 2A and Table S7). Of the “rare” pangenes shared by all four species, over 99% of pangenes were also conserved outside of the four species (Figure 2B and Table S8). On the other hand, 39–59% of the rare pangenes unique to only one of the four species were found in no additional species (Figure 2B and Table S8).

### Shared “rare” pangenes are more adapted to the codon usage of their host genomes

**Figure 3:**
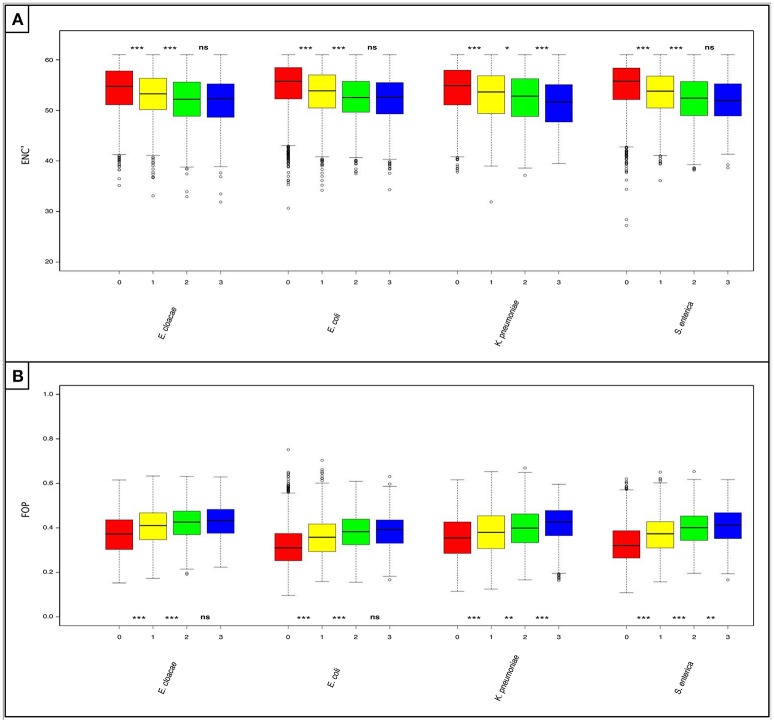
Horizontally acquired genes that are shared by more of the studied *Enterobacteriaceae* species are better adapted to the codon usage of their host species. Boxplots depicting: **(A)** levels of codon usage bias (as measured using ENC') and **(B)** frequency of the optimal codons (FOP) in the “rare” pangenes. For each species four boxes are given: red—“rare” pangenes that are not shared in any of the three other species; yellow—“rare” pangenes shared by one additional species; green—“rare” pangenes shared by two additional species; blue—“rare” pangenes shared by all species. Whisker length for each boxplot represents 1.5 IQR. Statistical significance of differences between the gene-loss groups according to a non-paired, one-sided Mann-Whitney-Wilcoxon test is denoted by: ^***^P ≤ 0.001, ^**^P ≤ 0.01, ^*^P ≤ 0.05, and (ns) for *P*>0.05.

We analyzed the codon usage patterns of the shared and non-shared “rare” pangenes. To do so, we used two metrices, ENC' and FoP. ENC' estimates overall levels of codon bias (Novembre, 2002), while FoP examines the tendency of genes to use a specific set of “optimal” codons that are predicted to be more favored in a species of interest (Ikemura, 1981). When ENC' was used, we found that ‘rare’ pangenes unique to each species are significantly less biased in their codon usage (higher ENC' values) than “rares” shared by one additional closely-related species, which in turn are significantly less biased than the “rare” pangenes shared by two additional species (*P* < 0.05, Figure 3A and Table S9). At the same time, statistically significant differences in the codon usage bias between the “rare” pangenes shared by two additional closely-related species and the “rares” shared by all analyzed species were observed only in *K. pneumoniae* (Figure 3A and Table S9).

When examining FoP, we found that “rare” pangenes unique to each species use significantly less optimized codons than “rares” shared by one additional closely-related species, which in turn use significantly less optimized codons than the “rare” pangenes shared by two additional closely-related species (*P* < 0.0025, Figure 3B and Table S10). “Rare” pangenes shared by all closely-related species had significantly higher frequencies of optimal codons than the “rares” shared by only three species only in *K. pneumoniae* and *S. enterica* (*P* < 0.007, Figure 3B and Table S10).

## Discussion

We show that within the *Enterobacteriaceae* family a large fraction of horizontally acquired genes are shared between species (38–51%, depending on the species in question). Among these shared genes, 59.5–68.8% have at least 75% sequence identity with their homologs in the other studied species. This indicates that a high proportion of the shared acquired genes were transferred from close relatives of the studied species or may even represent cases of gene transfer between the studied species. Our saturation analyses indicated that we are likely under-estimating the proportion of horizontally acquired genes that are shared between species, and that with the sequencing of additional genomes more such genes would be identified. At the same time, we do identify significant differences in conservation and codon usage between the horizontally acquired genes we found to be shared by more species, and those we found to be shared by less species. The existence of such differences indicates that there is indeed variation in the tendency of different genes to be shared and that the variation we observe in the extent of sharing is not solely due to limitations in data availability.

Why would some horizontally acquired genes be more frequently shared than others? One possibility is that the function of genes will affect their propensity of being transferred. Unfortunately, many of the “rare,” horizontally acquired pangenes do not carry a known annotated function (Table S11). It is therefore not possible to compare in an informative manner the functions of horizontally acquired genes shared by more species to those shared by less species. More research will be needed to understand which gene functions are more ubiquitously acquired horizontally.

Another factor that may increase the chances of finding certain genes in a higher number of studied species is their compatibility with the genome of their new host. We show that genes found in a higher number of the studied species tend to be more biased in their codon usage and are encoded by more optimized codons. Expression of acquired genes that are more compatible with the codon usage patterns of the host is expected to incur lower fitness costs (Angov, 2011; Plotkin and Kudla, 2011; Tuller et al., 2011; Park and Zhang, 2012), thus increasing the chances that they would be maintained within their host species for longer periods. Since the four closely related studied species have an almost identical set of optimal codons (Table S5), genes optimized for expression in one of the species are also optimized for expression in the other species. This in turn may make it easier for genes that are more optimized in their codon usage in one of these species to be maintained within the genome of another species once it is acquired.

The age of genes and the amount of time they have been present within our group of species may also affect their chances of being shared across the studied species, with older genes having more time to be shared than younger ones. We found that horizontally acquired genes unique to only one species tended to be shorter than those shared by an additional species (Table S12). A correlation between gene length and age has been observed in eukaryotic organisms (Grishkevich and Yanai, 2014; Schlotterer, 2015). If gene length is also indicative of age in bacteria, it may suggest that some of the genes unique to only one of the studied species are relatively young and didn't have as much time to be acquired by additional species. Finally, it is also possible that there is a rich-get-richer type of process at play. Genes that are present in a higher number of species are more likely to be horizontally transferred into additional species, simply because such genes can be acquired from a larger number of sources.

Among the “rare” pangenes that were found to be unique to only one of the four studied species, a large fraction was also not found in any other genome contained within the NCBI databases (between 39.23 and 59.07% for the four species, see Table S8). Such genes with no known homologs in any other species, termed ORFans in prokaryotes, are a known phenomenon, but their origins remain unclear. Some of these genes may represent instances of gene transfer from bacteria whose genomes are yet to be sequenced or events of gene transfer from non-bacterial genomes (Daubin and Ochman, 2004; Cortez et al., 2009; Schlotterer, 2015). However, it is also possible that these genes may not be horizontally acquired altogether and represent *de novo* created genes formed within their host genome (Tautz and Domazet-Loso, 2011; Fellner et al., 2015; Schlotterer, 2015) or some annotation artifacts, resulting from the mis-annotation of a non-genic sequence as a gene. Supporting the notion that genes we identified as putative ORFans are not primarily an artifact of our bacterial genome database size and breadth, we found that they have different properties from the non-ORFan genes unique to only one of the studied species. Consistently with the previous studies, ORFans have, on average, shorter gene length than the non-ORFan “unique” genes (see refs. Daubin and Ochman, 2004; Yu and Stoltzfus, 2012; Tatarinova et al., 2016 and Table S13). Also, these genes tend to be even less biased in their codon usage and have a smaller fraction of optimal codons in their gene sequences than non-ORFan unique genes (Table S13).

To conclude, our results reveal extensive sharing of horizontally acquired genes between closely related bacterial species. These results demonstrate the existence of a large pool of frequently horizontally acquired genes, displaying distinct conservation and codon usage patterns, when compared to horizontally acquired genes that are less frequently shared.

## Author contributions

EB participated in the study's design, carried out the analyses and co-wrote the manuscript. RH conceived the study, participated in its design, supervised the analyses, and co-wrote the manuscript. Both authors read and approved the final manuscript.

### Conflict of interest statement

The authors declare that the research was conducted in the absence of any commercial or financial relationships that could be construed as a potential conflict of interest.
