# Supplementary material for: Horizontally Acquired Genes Are Often Shared between Closely Related Bacterial Species
Source: Front Microbiol. 2017 Aug 25;8:1536. doi: 10.3389/fmicb.2017.01536 (PMC5575156; doi:10.3389/fmicb.2017.01536)
Supplement: Supplementary file 1 [file Table1.DOC]

**Table S1. Bacterial strains analyzed in the study**

| ***Enterobacter cloacae*** | |
| --- | --- |
| NZ_CP010384 | *Enterobacter cloacae* str. 34399 |
| NZ_CP010376 | *Enterobacter cloacae* str. 34977 |
| NZ_CP012165 | *Enterobacter cloacae* str. 34978 |
| NZ_CP010377 | *Enterobacter cloacae* str. 34983 |
| NZ_CP012167 | *Enterobacter cloacae* str. 34998 |
| NC_014121 | *Enterobacter cloacae* subsp. cloacae str. ATCC 13047 |
| NZ_CP011572 | *Enterobacter cloacae* str. CAV1311 |
| NZ_CP011581 | *Enterobacter cloacae* str. CAV1411 |
| NZ_CP011584 | *Enterobacter cloacae* str. CAV1668 |
| NZ_CP011650 | *Enterobacter cloacae* str. CAV1669 |
| NZ_CP008823 | *Enterobacter cloacae* str. ECNIH2 |
| NZ_CP008897 | *Enterobacter cloacae* str. ECNIH3 |
| NZ_CP009850 | *Enterobacter cloacae* str. ECNIH4 |
| NZ_CP009854 | *Enterobacter cloacae* str. ECNIH5 |
| NZ_CP008905 | *Enterobacter cloacae* str. ECR091 |
| NC_016514 | *Enterobacter cloacae* str. EcWSU1 |
| NC_018405 | *Enterobacter cloacae* subsp. cloacae str. ENHKU01 |
| NZ_CP009756 | *Enterobacter cloacae* str. GGT036 |
| NZ_CP006580 | *Enterobacter cloacae* str. P101 |
| NC_018079 | *Enterobacter cloacae* subsp. dissolvens str. SDM |
| NZ_CP011798 | *Enterobacter cloacae* str. UW5 |
| ***Escherichia coli*** | |
| NC_017626 | *Escherichia coli* str. 042 |
| NC_013364 | *Escherichia coli* O111 H- str. 11128 |
| NC_013361 | *Escherichia coli* O26 H11 str. 11368 |
| NC_013353 | *Escherichia coli* O103 H2 str. 12009 |
| NZ_CP009166 | *Escherichia coli* str. 1303 |
| NC_017328 | *Shigella flexneri* str. 2002017 |
| NZ_CP004056 | *Shigella flexneri* str. 2003036 |
| NC_018650 | *Escherichia coli* O104 H4 str. 2009EL-2050 |
| NC_018661 | *Escherichia coli* O104 H4 str. 2009EL-2071 |
| NC_018658 | *Escherichia coli* O104 H4 str. 2011C-3493 |
| NC_004741 | *Shigella flexneri* 2a str. 2457T |
| NC_004337 | *Shigella flexneri* 2a str. 301 |
| NC_008253 | *Escherichia coli* str. 536 |
| NC_016822 | *Shigella sonnei* str. 53G |
| NC_011748 | *Escherichia coli* str. 55989 |
| NZ_CP010371 | *Escherichia coli* str. 6409 |
| NZ_CP010315 | *Escherichia coli* str. 789 |
| NC_008258 | *Shigella flexneri* 5 str. 8401 |
| NZ_CP009106 | *Escherichia coli* str. 94-3024 |
| NC_017631 | *Escherichia coli* str. ABU 83972 |
| NZ_CP007442 | *Escherichia coli* str. ACN001 |
| NZ_CP005930 | *Escherichia coli* str. APEC IMT5155 |
| NC_008563 | *Escherichia coli* str. APEC O1 |
| NC_020163 | *Escherichia coli* str. APEC O78 |
| NZ_CP009072 | *Escherichia coli* str. ATCC 25922 |
| NC_010468 | *Escherichia coli* str. ATCC 8739 |
| NZ_CP011511 | *Shigella boydii* str. ATCC 9210 |
| NC_012967 | *Escherichia coli* B str. REL606 |
| NZ_CP010816 | *Escherichia coli* B str. BL21 |
| NC_010658 | *Shigella boydii* CDC 3083-94 str. BS512 |
| NZ_CP011331 | *Escherichia coli* O104 H4 str. C227-11 |
| NC_013941 | *Escherichia coli* O55 H7 str. CB9615 |
| NC_017646 | *Escherichia coli* O7 K1 str. CE10 |
| NZ_CP011416 | *Escherichia coli* str. CFSAN029787 |
| NC_004431 | *Escherichia coli* str. CFT073 |
| NZ_CP011018 | *Escherichia coli* str. CI5 |
| NC_017652 | *Escherichia coli* str. clone D i14 |
| NZ_CP013253 | *Escherichia coli* str. CQSW20 |
| NC_017625 | *Escherichia coli* str. DH1 |
| NC_011601 | *Escherichia coli* O127 H6 str. E2348/69 |
| NC_009801 | *Escherichia coli* O139 H28 str. E24377A |
| NC_011353 | *Escherichia coli* O157 H7 str. EC4115 |
| NZ_CP010344 | *Escherichia coli* str. ECC-1470 |
| NZ_CP009859 | *Escherichia coli* str. ECONIH1 |
| NZ_CP008957 | *Escherichia coli* O157 H7 str. EDL933 |
| NC_017633 | *Escherichia coli* str. ETEC H10407 |
| NZ_CP007037 | *Shigella flexneri* str. G1663 |
| NC_009800 | *Escherichia coli* str. HS |
| NZ_HF572917 | *Escherichia coli* str. HUSEC2011 |
| NC_011741 | *Escherichia coli* str. IAI1 |
| NC_011750 | *Escherichia coli* str. IAI39 |
| NC_017628 | *Escherichia coli* str. IHE3034 |
| NC_022648 | *Escherichia coli* str. JJ1886 |
| NZ_LN832404 | *Escherichia coli* str. K-12 substr. AG100 |
| NZ_CP009644 | *Escherichia coli* str. K-12 substr. ER2796 |
| NZ_CP009273 | *Escherichia coli* str. K-12 substr. BW25113 |
| NC_000913 | *Escherichia coli* str. K-12 substr. MG1655 |
| NC_007779 | *Escherichia col* str. K-12 substr. W3110 |
| NZ_LM995446 | *Escherichia coli* str. K-12 substr. RV308 |
| NZ_CP011495 | *Escherichia coli* str. K-12 substr. NCM3722 |
| NZ_CP008801 | *Escherichia coli* str. KLY |
| NC_011993 | *Escherichia coli* str. LF82 |
| NC_022364 | *Escherichia coli* str. LY180 |
| NZ_CP010876 | *Escherichia coli* str. MNCRE44 |
| NC_017644 | *Escherichia coli* str. NA114 |
| NZ_LM651928 | *Shigella flexneri* str. NCTC1 |
| NZ_CP007799 | *Escherichia coli* str. Nissle 1917 |
| NC_017634 | *Escherichia coli* O83 H1 str. NRG 857C |
| NC_017663 | *Escherichia coli* str. P12b |
| NZ_CP006632 | *Escherichia coli* str. PCN033 |
| NZ_CP006636 | *Escherichia coli* str. PCN061 |
| NC_022370 | *Escherichia coli* str. PMV-1 |
| NC_017656 | *Escherichia coli* O55 H7 str. RM12579 |
| NZ_CP007136 | *Escherichia coli* O145 H28 str. RM12581 |
| NZ_CP007133 | *Escherichia coli* O145 H28 str. RM12761 |
| NZ_CP006027 | *Escherichia coli* O145 H28 str. RM13514 |
| NZ_CP006262 | *Escherichia coli* O145 H28 str. RM13516 |
| NZ_CP009104 | *Escherichia coli* str. RM9387 |
| NZ_CP011113 | *Escherichia coli* str. RR1 |
| NC_002695 | *Escherichia coli* O157 H7 str. Sakai substr. RIMD 0509952 |
| NZ_CP007592 | *Escherichia coli* O157 H16 str. Santai |
| NC_007613 | *Shigella boydii*str. Sb227 |
| NC_007606 | *Shigella dysenteriae* str. Sd197 |
| NC_011415 | *Escherichia coli* str. SE11 |
| NC_013654 | *Escherichia coli* str. SE15 |
| NZ_CP007594 | *Escherichia coli* str. SEC470 |
| NZ_CP012631 | *Escherichia coli* str. SF-173 |
| NZ_CP004057 | *Shigella flexneri* str. Shi06HN006 |
| NC_010498 | *Escherichia coli* str. SMS-3-5 |
| NC_007384 | *Shigella sonnei* str. Ss046 |
| NZ_CP008805 | *Escherichia coli* O157 H7 str. SS17 |
| NZ_CP010304 | *Escherichia coli* O157 H7 str. SS52 |
| NZ_HG941718 | *Escherichia coli* str. ST131 |
| NZ_CP007392 | *Escherichia coli* str. ST2747 |
| NZ_CP007265 | *Escherichia coli* str. ST540 |
| NC_013008 | *Escherichia coli* O157 H7 str. TW14359 |
| NC_017632 | *Escherichia coli* str. UM146 |
| NC_017641 | *Escherichia coli* str. UMNK88 |
| NC_007946 | *Escherichia coli* str. UTI89 |
| NZ_CP011134 | *Escherichia coli* str. VR50 |
| NC_017635 | *Escherichia coli* str. W |
| NC_017906 | *Escherichia coli* str. Xuzhou21 |
| NZ_CP013112 | *Escherichia coli* str. YD786 |
| ***Klebsiella pneumoniae*** | |
| NC_018522 | *Klebsiella pneumoniae* subsp. pneumoniae str. 1084 |
| NZ_CP006722 | *Klebsiella pneumoniae* subsp. pneumoniae str. 1158 |
| NZ_CP011313 | *Klebsiella pneumoniae* subsp. pneumoniae str. 234-12 |
| NZ_CP006923 | *Klebsiella pneumoniae* str. 30660/NJST258_1 |
| NZ_CP006918 | *Klebsiella pneumoniae* str. 30684/NJST258_2 |
| NZ_CP010361 | *Klebsiella pneumoniae* str. 32192 |
| NC_011283 | *Klebsiella pneumoniae* str. 342 |
| NZ_CP010392 | *Klebsiella pneumoniae* str. 34618 |
| NZ_CP011980 | *Klebsiella pneumoniae* str. 500_1420 |
| NZ_CP009208 | *Klebsiella pneumoniae* subsp. pneumoniae str. ATCC 43816 KPPR1 |
| NC_009648 | *Klebsiella pneumoniae* subsp. pneumoniae MGH 78578 str. ATCC 700721 |
| NZ_CP009114 | *Klebsiella pneumoniae* str. blaNDM-1 |
| NZ_CP011624 | *Klebsiella pneumoniae* str. CAV1344 |
| NZ_CP011578 | *Klebsiella pneumoniae* str. CAV1392 |
| NZ_CP011647 | *Klebsiella pneumoniae* str. CAV1596 |
| NC_022566 | *Klebsiella pneumoniae* str. CG43 |
| NZ_CP011976 | *Klebsiella pneumoniae* str. DMC1097 |
| NZ_CP006738 | *Klebsiella pneumoniae* str. HK787 |
| NZ_CP012300 | *Klebsiella pneumoniae* subsp. pneumoniae str. HKUOPLC |
| NC_016845 | *Klebsiella pneumoniae* subsp. pneumoniae HS11286 str. HS11286 |
| NZ_CP013711 | *Klebsiella pneumoniae* str. J1 |
| NC_022082 | *Klebsiella pneumoniae* str. JM45 |
| NC_017540 | *Klebsiella pneumoniae* str. KCTC 2242 |
| NZ_CP003999 | *Klebsiella pneumoniae* subsp. pneumoniae str. Kp13 |
| NZ_FO834906 | *Klebsiella pneumoniae* str. Kp52.145 |
| NZ_CP008827 | *Klebsiella pneumoniae* subsp. pneumoniae str. KPNIH1 |
| NZ_CP007727 | *Klebsiella pneumoniae* subsp. pneumoniae str. KPNIH10 |
| NZ_CP008797 | *Klebsiella pneumoniae* subsp. pneumoniae str. KPNIH24 |
| NZ_CP007731 | *Klebsiella pneumoniae* subsp. pneumoniae str. KPNIH27 |
| NZ_CP009863 | *Klebsiella pneumoniae* subsp. pneumoniae str. KPNIH29 |
| NZ_CP009872 | *Klebsiella pneumoniae* subsp. pneumoniae str. KPNIH30 |
| NZ_CP009876 | *Klebsiella pneumoniae* subsp. pneumoniae str. KPNIH31 |
| NZ_CP009775 | *Klebsiella pneumoniae* subsp. pneumoniae str. KPNIH32 |
| NZ_CP009771 | *Klebsiella pneumoniae* subsp. pneumoniae str. KPNIH33 |
| NZ_CP008831 | *Klebsiella pneumoniae* subsp. pneumoniae str. KPR0928 |
| NC_012731 | *Klebsiella pneumoniae* subsp. pneumoniae str. NTUH-K2044 |
| NZ_CP006798 | *Klebsiella pneumoniae* subsp. pneumoniae str. PittNDM01 |
| NZ_CP008929 | *Klebsiella pneumoniae* str. PMK1 |
| NZ_CP011985 | *Klebsiella pneumoniae* str. UHKPC07 |
| NZ_CP011989 | *Klebsiella pneumoniae* str. UHKPC33 |
| NZ_CP009461 | *Klebsiella pneumoniae* str. XH209 |
| ***Salmonella enterica*** | |
| NZ_CP007534 | *Salmonella enterica* subsp. enterica serovar Abony str. 0014 |
| NC_021820 | *Salmonella enterica* subsp. enterica serovar 4,[5],12 i - str. 08-1736 |
| NZ_LN649235 | *Salmonella enterica* subsp. enterica serovar Infantis str. 1326/28 |
| NZ_CP007581 | *Salmonella enterica* subsp. enterica serovar Typhimurium str. 138736 |
| NC_016856 | *Salmonella enterica* subsp. enterica serovar Typhimurium str. 14028S |
| NZ_CP011394 | *Salmonella enterica* subsp. enterica serovar Enteritidis str. 18569 |
| NC_022991 | *Salmonella enterica* subsp. enterica serovar Agona str. 24249 |
| NC_011274 | *Salmonella enterica* subsp. enterica serovar Gallinarum str. 287/91 |
| NZ_CP012681 | *Salmonella enterica* subsp. enterica serovar Typhimurium str. 33676 |
| NC_021810 | *Salmonella enterica* subsp. enterica serovar Heidelberg str. 41578 |
| NZ_CP011259 | *Salmonella enterica* subsp. enterica serovar Agona str. 460004 2-1 |
| NZ_CP007530 | *Salmonella enterica* subsp. enterica serovar Montevideo str. 507440-20 |
| NZ_CP009049 | *Salmonella enterica* subsp. enterica serovar Paratyphi A str. 50973 |
| NZ_CP007598 | *Salmonella enterica* subsp. enterica serovar Enteritidis str. 77-1427 |
| NC_017046 | *Salmonella enterica* subsp. enterica serovar Typhimurium str. 798 |
| NC_011147 | *Salmonella enterica* subsp. enterica serovar Paratyphi A str. AKU_12601 |
| NZ_LN890518 | *Salmonella enterica* subsp. enterica serovar Weltevreden str. 10259 |
| NZ_LN890520 | *Salmonella enterica* subsp. enterica serovar Weltevreden str. C2346 |
| NZ_CP012344 | *Salmonella enterica* subsp. enterica serovar Choleraesuis str. ATCC 10708 |
| NZ_CP009102 | *Salmonella enterica* subsp. enterica serovar Typhimurium str. ATCC 13311 |
| NZ_CP012349 | *Salmonella enterica* subsp. enterica serovar Sloterdijk str. ATCC 15791 |
| NZ_CP007532 | *Salmonella enterica* subsp. enterica serovar Abaetetuba str. ATCC 35640 |
| NZ_CP012346 | *Salmonella enterica* subsp. enterica serovar Panama str. ATCC 7378 |
| NZ_CP011396 | *Salmonella enterica* subsp. enterica serovar Thompson str. ATCC 8391 |
| NZ_CP012347 | *Salmonella enterica* subsp. enterica serovar Pullorum str. ATCC 9120 |
| NC_006511 | *Salmonella enterica* subsp. enterica serovar Paratyphi A str. ATCC 9150 |
| NZ_CP007531 | *Salmonella enterica* subsp. enterica serovar Anatum str. ATCC BAA-1592 |
| NC_017623 | *Salmonella enterica* subsp. enterica serovar Heidelberg str. B182 |
| NZ_CP007639 | *Salmonella enterica* subsp. enterica serovar Choleraesuis str. C500 |
| NZ_CP007211 | *Salmonella enterica* subsp. enterica serovar Anatum str. CDC 06-0532 |
| NZ_CP007559 | *Salmonella enterica* subsp. enterica serovar Newport str. CDC 2010K-2159 |
| NZ_CP007523 | *Salmonella enterica* subsp. enterica serovar Typhimurium str. CDC 2011K-0870 |
| NZ_CP007528 | *Salmonella enterica* subsp. enterica serovar Enteritidis str. CDC_2010K_0968 |
| NC_022221 | *Salmonella enterica* subsp. enterica serovar Gallinarum/pullorum str. CDC1983-67 |
| NC_021844 | *Salmonella enterica* subsp. enterica serovar Bareilly str. CFSAN000189 str. CFSAN000189 |
| NZ_CP007533 | *Salmonella enterica* subsp. enterica serovar Bredeney str. CFSAN001080 |
| NC_021814 | *Salmonella enterica* subsp. enterica serovar Typhimurium var. 5- str. CFSAN001921 |
| NC_020307 | *Salmonella enterica* subsp. enterica serovar Javiana str. CFSAN001992 |
| NC_021818 | *Salmonella enterica* subsp. enterica serovar Cubana str. CFSAN002050 |
| NZ_CP005995 | *Salmonella enterica* subsp. enterica serovar Heidelberg str. CFSAN002064 |
| NC_021812 | *Salmonella enterica* subsp. enterica serovar Heidelberg str. CFSAN002069 |
| NZ_CP009559 | *Salmonella enterica* subsp. enterica serovar Paratyphi A str. CMCC 50503 |
| NC_011205 | *Salmonella enterica* subsp. enterica serovar Dublin str. CT_02021853 |
| NC_003198 | *Salmonella enterica* subsp. enterica serovar Typhi str. CT18 |
| NZ_CP010282 | *Salmonella enterica* subsp. enterica serovar Newport str. CVM 21538 str. CVM 21538 |
| NZ_CP010283 | *Salmonella enterica* subsp. enterica serovar Newport str. CVM 21550 |
| NZ_CP010279 | *Salmonella enterica* subsp. enterica serovar Newport str. CVM 22425 |
| NZ_CP010280 | *Salmonella enterica* subsp. enterica serovar Newport str. CVM 22462 |
| NZ_CP010281 | *Salmonella enterica* subsp. enterica serovar Newport str. CVM 22513 |
| NZ_CP010284 | *Salmonella enterica* subsp. enterica serovar Newport str. CVM N1543 |
| NZ_CP009561 | *Salmonella enterica* subsp. enterica serovar Newport str. CVM N18486 |
| NC_011094 | *Salmonella enterica* subsp. enterica serovar Schwarzengrund str. CVM19633 |
| NC_016854 | *Salmonella enterica* subsp. enterica serovar Typhimurium str. D23580 |
| NC_022569 | *Salmonella enterica* subsp. enterica serovar Typhimurium str. DT104 |
| NC_022544 | *Salmonella enterica* subsp. enterica serovar Typhimurium str. DT2 |
| NZ_CP007507 | *Salmonella enterica* subsp. enterica serovar Enteritidis str. Durban |
| NZ_CP007320 | *Salmonella enterica* subsp. enterica serovar Enteritidis str. EC20090135 |
| NZ_CP007321 | *Salmonella enterica* subsp. enterica serovar Enteritidis str. EC20090193 |
| NZ_CP007322 | *Salmonella enterica* subsp. enterica serovar Enteritidis str. EC20090332 |
| NZ_CP007422 | *Salmonella enterica* subsp. enterica serovar Enteritidis str. EC20090531 |
| NZ_CP007249 | *Salmonella enterica* subsp. enterica serovar Enteritidis str. EC20090641 |
| NZ_CP007248 | *Salmonella enterica* subsp. enterica serovar Enteritidis str. EC20090698 |
| NZ_CP007421 | *Salmonella enterica* subsp. enterica serovar Enteritidis str. EC20090884 |
| NZ_CP007246 | *Salmonella enterica* subsp. enterica serovar Enteritidis str. EC20100101 |
| NZ_CP007420 | *Salmonella enterica* subsp. enterica serovar Enteritidis str. EC20100103 |
| NZ_CP007358 | *Salmonella enterica* subsp. enterica serovar Enteritidis str. EC20100130 |
| NZ_CP007359 | *Salmonella enterica* subsp. enterica serovar Enteritidis str. EC20100134 |
| NZ_CP007247 | *Salmonella enterica* subsp. enterica serovar Enteritidis str. EC20110221 |
| NZ_CP007266 | *Salmonella enterstrainica* subsp. enterica serovar Enteritidis str. EC20110223 |
| NZ_CP007251 | *Salmonella enterica* subsp. enterica serovar Enteritidis str. EC20110353 |
| NZ_CP007175 | *Salmonella enterica* subsp. enterica serovar Enteritidis str. EC20110354 |
| NZ_CP007250 | *Salmonella enterica* subsp. enterica serovar Enteritidis str. EC20110355 |
| NZ_CP007262 | *Salmonella enterica* subsp. enterica serovar Enteritidis str. EC20110356 |
| NZ_CP007261 | *Salmonella enterica* subsp. enterica serovar Enteritidis str. EC20110357 |
| NZ_CP007260 | *Salmonella enterica* subsp. enterica serovar Enteritidis str. EC20110358 |
| NZ_CP007259 | *Salmonella enterica* subsp. enterica serovar Enteritidis str. EC20110359 |
| NZ_CP007258 | *Salmonella enterica* subsp. enterica serovar Enteritidis str. EC20110360 |
| NZ_CP007263 | *Salmonella enterica* subsp. enterica serovar Enteritidis str. EC20110361 |
| NZ_CP007254 | *Salmonella enterica* subsp. enterica serovar Enteritidis str. EC20111095 |
| NZ_CP007253 | *Salmonella enterica* subsp. enterica serovar Enteritidis str. EC20111174 |
| NZ_CP007252 | *Salmonella enterica* subsp. enterica serovar Enteritidis str. EC20111175 |
| NZ_CP007329 | *Salmonella enterica* subsp. enterica serovar Enteritidis str. EC20120002 |
| NZ_CP007267 | *Salmonella enterica* subsp. enterica serovar Enteritidis str. EC20120005 |
| NZ_CP007245 | *Salmonella enterica* subsp. enterica serovar Enteritidis str. EC20120008 |
| NZ_CP007332 | *Salmonella enterica* subsp. enterica serovar Enteritidis str. EC20120916 |
| NZ_CP007463 | *Salmonella enterica* subsp. enterica serovar Enteritidis str. EC20120929 |
| NZ_CP011365 | *Salmonella enterica* subsp. enterica serovar Typhimurium str. FORC_015 |
| NZ_CP012144 | *Salmonella enterica* subsp. enterica serovar Typhimurium str. FORC_020 |
| NZ_AP014565 | *Salmonella enterica* subsp. enterica serovar Typhimurium str. L-3553 |
| NC_003197 | *Salmonella enterica* subsp. enterica serovar Typhimurium str. LT2 |
| NZ_CP009083 | *Salmonella enterica* subsp. enterica serovar Enteritidis str. OLF-SE1-1019-1 |
| NZ_CP009092 | *Salmonella enterica* subsp. enterica serovar Enteritidis str. OLF-SE10-10052 |
| NZ_CP009093 | *Salmonella enterica* subsp. enterica serovar Enteritidis str. OLF-SE11-10058 |
| NZ_CP009084 | *Salmonella enterica* subsp. enterica serovar Enteritidis str. OLF-SE2-98984-6 |
| NZ_CP009085 | *Salmonella enterica* subsp. enterica serovar Enteritidis str. OLF-SE3-98983-4 |
| NZ_CP009086 | *Salmonella enterica* subsp. enterica serovar Enteritidis str. OLF-SE4-0317-8 |
| NZ_CP009087 | *Salmonella enterica* subsp. enterica serovar Enteritidis str. OLF-SE5-1104-2 |
| NZ_CP009088 | *Salmonella enterica* subsp. enterica serovar Enteritidis str. OLF-SE6-00219-16 |
| NZ_CP009089 | *Salmonella enterica* subsp. enterica serovar Enteritidis str. OLF-SE7-100819 |
| NZ_CP009090 | *Salmonella enterica* subsp. enterica serovar Enteritidis str. OLF-SE8-1021710 |
| NZ_CP009091 | *Salmonella enterica* subsp. enterica serovar Enteritidis str. OLF-SE9-10012 |
| NC_016832 | *Salmonella enterica* subsp. enterica serovar Typhi str. P-stx-12 |
| NC_011294 | *Salmonella enterica* subsp. enterica serovar Enteritidis str. P125109 |
| NZ_CP006693 | *Salmonella enterica* subsp. arizonae serovar 62 z36 - str. RKS2983 |
| NC_012125 | *Salmonella enterica* subsp. enterica serovar Paratyphi C str. RKS4594 |
| NC_016831 | *Salmonella enterica* subsp. enterica serovar Gallinarum/pullorum str. RKS5078 |
| NZ_CP012513 | *Salmonella enterica* subsp. enterica serovar Thompson str. RM1984 |
| NZ_CP012514 | *Salmonella enterica* subsp. enterica serovar Thompson str. RM1986 |
| NC_022525 | *Salmonella enterica* subsp. enterica serovar Thompson str. RM6836 |
| NC_010067 | *Salmonella enterica* subsp. arizonae serovar 62 z4,z23 - str. RSK2980 |
| NC_021984 | *Salmonella enterica* subsp. enterica serovar Pullorum str. S06004 |
| NZ_CP007274 | *Salmonella enterica* subsp. enterica serovar Enteritidis str. SA20093266 |
| NC_006905 | *Salmonella enterica* subsp. enterica serovar Choleraesuis str. SC-B67 |
| NZ_CP011790 | *Salmonella enterica* subsp. enterica serovar Enteritidis str. SEE1 |
| NZ_CP011791 | *Salmonella enterica* subsp. enterica serovar Enteritidis str. SEE2 |
| NZ_CP008928 | *Salmonella enterica* subsp. enterica serovar Enteritidis str. SEJ |
| NC_016810 | *Salmonella enterica* subsp. enterica serovar Typhimurium str. SL1344 |
| NC_011080 | *Salmonella enterica* subsp. enterica serovar Newport str. SL254 |
| NC_011083 | *Salmonella enterica* subsp. enterica serovar Heidelberg str. SL476 |
| NC_011149 | *Salmonella enterica* subsp. enterica serovar Agona str. SL483 |
| NC_010102 | *Salmonella enterica* subsp. enterica serovar Paratyphi B str. SPB7 |
| NC_016857 | *Salmonella enterica* subsp. enterica serovar Typhimurium str. ST4/74 |
| NC_016860 | *Salmonella enterica* subsp. enterica serovar Typhimurium str. T000240 |
| NZ_CP007505 | *Salmonella enterica* subsp. enterica serovar Tennessee str. TXSC_TXSC08-19 |
| NC_004631 | *Salmonella enterica* subsp. enterica serovar Typhi str. Ty2 |
| NC_021151 | *Salmonella enterica* subsp. enterica serovar Typhimurium str. U288 |
| NC_016863 | *Salmonella enterica* subsp. enterica serovar Typhimurium str. UK-1 |
| NZ_CP007483 | *Salmonella enterica* subsp. enterica serovar Anatum str. USDA-ARS-USMARC-1175 |
| NZ_CP007584 | *Salmonella enterica* subsp. enterica serovar Anatum str. USDA-ARS-USMARC-1735 |
| NZ_CP007235 | *Salmonella enterica* subsp. enterica serovar Typhimurium str. USDA-ARS-USMARC-1899 |
| NZ_CP007222 | *Salmonella enterica* subsp. enterica serovar Montevideo str. USDA-ARS-USMARC-1903 |
| NZ_CP007540 | *Salmonella enterica* subsp. enterica serovar Montevideo str. USDA-ARS-USMARC-1921 |
| NZ_CP007216 | *Salmonella enterica* subsp. enterica serovar Newport str. USDA-ARS-USMARC-1927 |
| NC_021902 | *Salmonella enterica* subsp. enterica serovar Newport str. USMARC-S3124.1 |
| NZ_CP011428 | *Salmonella enterica* subsp. enterica str. YU39 |
