# Supplementary material for: Horizontally Acquired Genes Are Often Shared between Closely Related Bacterial Species
Source: Front Microbiol. 2017 Aug 25;8:1536. doi: 10.3389/fmicb.2017.01536 (PMC5575156; doi:10.3389/fmicb.2017.01536)
Supplement: Supplementary file 2 [file Table2.DOC]

**Table S2.** List of strains removed from the further analysis

| ***Enterobacter cloacae*** | |
| --- | --- |
| No removed strains | |
| ***Escherichia coli*** | |
| NC_012947 | *Escherichia coli* str. BL21-Gold(DE3)pLysS AG |
| NC_012892 | *Escherichia coli* str. BL21(DE3) |
| NC_012971 | *Escherichia coli* str. BL21(DE3) |
| NZ_CP010585 | *Escherichia coli* C41(DE3) |
| NZ_CP011938 | *Escherichia coli* str. C43(DE3) |
| NC_017651 | *Escherichia coli* str. clone D i2 |
| NC_017638 | *Escherichia coli* str. DH1 |
| NZ_CP012125 | *Escherichia coli* str. DH1Ec095 |
| NZ_CP012126 | *Escherichia coli* str. DH1Ec104 |
| NZ_CP012127 | *Escherichia coli* str. DH1Ec169 |
| NZ_CP011343 | *Escherichia coli* str. GM4792 |
| NZ_CP009789 | *Escherichia coli* str. K-12 ER3413 |
| NZ_CP010438 | *Escherichia coli* str. K-12 MG1655 substr. ER3454 |
| NZ_CP010440 | *Escherichia coli* str. K-12 MG1655 substr. ER3476 |
| NZ_CP010441 | *Escherichia coli* str. K-12 MG1655 substr. ER3445 |
| NZ_CP010442 | *Escherichia coli* str. K-12 MG1655 substr. ER3466 |
| NZ_CP010443 | *Escherichia coli* str. K-12 MG1655 substr. ER3446 |
| NZ_CP010444 | *Escherichia coli* str. K-12 MG1655 substr. ER3475 |
| NC_012759 | *Escherichia coli* str. K-12 substr. BW2952 |
| NC_010473 | *Escherichia coli* str. K-12 substr. DH10B |
| NC_020518 | *Escherichia coli* str. K-12 substr. MDS42 |
| NZ_LM993812 | *Escherichia coli* str. K-12 substr. HMS174 |
| NZ_AKBV01000001 | *Escherichia coli* str. K-12 substr. MG1655 |
| NZ_AKVX01000001 | *Escherichia coli* str. K-12 substr. MG1655 |
| NZ_CP009685 | *Escherichia coli* str. K-12 substr. MG1655 |
| NZ_CP012868 | *Escherichia coli* str. K-12 substr. MG1655 |
| NZ_CP012869 | *Escherichia coli* str. K-12 substr. MG1655_TMP32XR1 |
| NZ_CP012870 | *Escherichia coli* str. K-12 substr. MG1655_TMP32XR2 |
| NZ_HG738867 | *Escherichia coli* str. K-12 substr. MC4100 |
| NC_016902 | *Escherichia coli* KO11FL str. KO11 |
| NC_017660 | *Escherichia coli* KO11FL str. KO11FL |
| NZ_CP010439 | *Escherichia coli* str. K-12 MG1655 substr. ER3440 |
| NZ_CP010445 | *Escherichia coli* str. K-12 MG1655 substr. ER3435 |
| NZ_CP011324 | *Escherichia coli* str. SQ2203 |
| NZ_CP011320 | *Escherichia coli* str. SQ37 |
| NZ_CP011321 | *Escherichia coli* str. SQ88 |
| NZ_CP007393 | *Escherichia coli* str. ST2747-A |
| NZ_CP007394 | *Escherichia coli* str. ST2747-AN |
| NZ_CP007390 | *Escherichia coli* str. ST540-A |
| NZ_CP007391 | *Escherichia coli* str. ST540-AN |
| NZ_AGTD01000001 | *Escherichia coli* UMNF18 str. UMNF18 |
| NC_017664 | *Escherichia coli* W str. W |
| ***Klebsiella pneumoniae*** | |
| No removed strains | |
| ***Salmonella enterica*** | |
| NC_021176 | *Salmonella enterica* subsp. enterica serovar Typhi str. Ty21a |
| NZ_CP007804 | *Salmonella enterica* subsp. enterica serovar Typhimurium str. VNP20009 |
| NZ_CP007269 | *Salmonella enterica* subsp. enterica serovar Enteritidis str. EC20121175 |
