# Supplementary material for: Horizontally Acquired Genes Are Often Shared between Closely Related Bacterial Species
Source: Front Microbiol. 2017 Aug 25;8:1536. doi: 10.3389/fmicb.2017.01536 (PMC5575156; doi:10.3389/fmicb.2017.01536)
Supplement: Supplementary file 3 [file Table3.DOC]

**Table S3. Average amino acid identity (AAI) of orthologous proteins in the pangenomes of the investigated species.**

| **Organism** | *E. cloacae* | *E. coli* | *K. pneumoniae* | *S. enterica* |
| --- | --- | --- | --- | --- |
| *E. cloacae* |  | 78.27 | 79.12 | 79.81 |
| *E. coli* |  |  | 78.53 | 81.73 |
| *K. pneumoniae* |  |  |  | 78.60 |
| *S. enterica* |  |  |  |  |
