# Supplementary material for: Horizontally Acquired Genes Are Often Shared between Closely Related Bacterial Species
Source: Front Microbiol. 2017 Aug 25;8:1536. doi: 10.3389/fmicb.2017.01536 (PMC5575156; doi:10.3389/fmicb.2017.01536)
Supplement: Supplementary file 4 [file Table4.DOC]

**Table S4**. List of merged strains

| ***Enterobacter cloacae*** | | |
| --- | --- | --- |
| **# of clustered strains** | **Cluster representative ID** | **Clustered strains IDs** |
| 4 | NZ_CP011572 | NZ_CP011572, NZ_CP011581, NZ_CP011584, NZ_CP011650 |
| 3 | NZ_CP008897 | NZ_CP008897, NZ_CP008905, NZ_CP009854 |
| ***Escherichia coli*** | | |
| **# of clustered strains** | **Cluster representative ID** | **Clustered strains IDs** |
| 4 | NC_011353 | NC_011353, NC_013008, NZ_CP008805, NZ_CP010304 |
| 4 | NC_018650 | NC_018650, NC_018661, NZ_CP011331, NZ_HF572917 |
| 2 | NC_017633 | NC_017633, NZ_CP007594 |
| 2 | NZ_CP006262 | NZ_CP006262, NZ_CP007133 |
| 2 | NZ_CP006027 | NZ_CP006027, NZ_CP007136 |
| 2 | NC_017906 | NC_017906, NZ_CP008957 |
| 6 | NC_007779 | NC_007779, NC_017625, NZ_CP009273, NZ_CP011495, NZ_LM995446, NZ_LN832404 |
| 2 | NC_012967 | NC_012967, NZ_CP010816 |
| 2 | NC_022648 | NC_022648, NZ_HG941718 |
| 2 | NZ_CP004056 | NZ_CP004056, NZ_CP004057 |
| ***Klebsiella pneumoniae*** | | |
| **# of clustered strains** | **Cluster representative ID** | **Clustered strains IDs** |
| 2 | NZ_CP007727 | NZ_CP007727, NZ_CP008827 |
| 7 | NZ_CP008797 | NZ_CP008797, NZ_CP008831, NZ_CP009872, NZ_CP010361, NZ_CP011980, NZ_CP011985, NZ_CP011989 |
| ***Salmonella enterica*** | | |
| **# of clustered strains** | **Cluster representative ID** | **Clustered strains IDs** |
| 2 | NC_004631 | NC_004631, NC_016832 |
| 2 | NC_021812 | NC_021812, NZ_CP005995 |
| 2 | NZ_CP011790 | NZ_CP011790, NZ_CP011791 |
| 2 | NC_022569 | NC_022569, NZ_CP007581 |
| 2 | NC_011294 | NC_011294, NZ_CP011394 |
| 3 | NC_022525 | NC_022525, NZ_CP012513, NZ_CP012514 |
| 42 | NZ_CP007175 | NZ_CP007175, NZ_CP007245, NZ_CP007246, NZ_CP007247, NZ_CP007248, NZ_CP007249, NZ_CP007250, NZ_CP007252, NZ_CP007253, NZ_CP007254, NZ_CP007258, NZ_CP007259, NZ_CP007260, NZ_CP007261, NZ_CP007262, NZ_CP007263, NZ_CP007266, NZ_CP007267, NZ_CP007320, NZ_CP007321, NZ_CP007322, NZ_CP007329, NZ_CP007358, NZ_CP007359, NZ_CP007420, NZ_CP007421, NZ_CP007422, NZ_CP007463, NZ_CP007507, NZ_CP007528, NZ_CP007598, NZ_CP008928, NZ_CP009083, NZ_CP009084, NZ_CP009085, NZ_CP009086, NZ_CP009087, NZ_CP009088, NZ_CP009089, NZ_CP009090, NZ_CP009092, NZ_CP009093 |
| 4 | NC_006511 | NC_006511, NC_011147, NZ_CP009049, NZ_CP009559 |
| 4 | NC_011080 | NC_011080, NZ_CP010281, NZ_CP010283, NZ_CP010284 |
| 3 | NC_011149 | NC_011149, NC_022991, NZ_CP011259 |
| 2 | NZ_CP010279 | NZ_CP010279, NZ_CP010280 |
| 6 | NC_016810 | NC_016810, NC_016856, NC_016857, NC_016863, NC_017046, NC_022544 |
| 2 | NZ_CP007235 | NZ_CP007235, NZ_CP007523 |
