# Supplementary material for: Horizontally Acquired Genes Are Often Shared between Closely Related Bacterial Species
Source: Front Microbiol. 2017 Aug 25;8:1536. doi: 10.3389/fmicb.2017.01536 (PMC5575156; doi:10.3389/fmicb.2017.01536)
Supplement: Supplementary file 5 [file Table5.DOC]

**Table S5.** Optimal codons in the investigated bacterial species

| **Organism** | **Ala** | **Arg** | **Asn** | **Asp** | **Cys** | **Gln** | **Glu** | **Gly** | **His** | **Ile** | **Leu** | **Lys** | **Phe** |
| --- | --- | --- | --- | --- | --- | --- | --- | --- | --- | --- | --- | --- | --- |
| *E. cloacae* | GCG | CGT/CGC | AAC | GAC | TGC | CAG | GAA | GGC | CAC | ATC | CTG | AAA | TTC |
| *E. coli* | GCG | CGC | AAC | GAC | TGC | CAG | GAA | GGC | CAC | ATC | CTG | AAA | TTC |
| *K. pneumoniae* | GCG | CGC | AAC | GAC | TGC | CAG | GAA | GGC | CAC | ATC | CTG | AAA | TTC |
| *S. enterica* | GCG | CGC | AAC | GAC | TGC | CAG | GAA | GGC | CAC | ATC | CTG | AAA | TTC |
| **Organism** | **Pro** | **Ser** | **Thr** | **Tyr** | **Val** |  |  |  |  |  |  |  |  |
| *E. cloacae* | CCG | TCC | ACC | TAC | GTG |  |  |  |  |  |  |  |  |
| *E. coli* | CCG | AGC | ACC | TAC | GTG |  |  |  |  |  |  |  |  |
| *K. pneumoniae* | CCG | AGC | ACC | TAC | GTG |  |  |  |  |  |  |  |  |
| *S. enterica* | CCG | AGC | ACC | TAC | GTG |  |  |  |  |  |  |  |  |
