# Supplementary material for: Horizontally Acquired Genes Are Often Shared between Closely Related Bacterial Species
Source: Front Microbiol. 2017 Aug 25;8:1536. doi: 10.3389/fmicb.2017.01536 (PMC5575156; doi:10.3389/fmicb.2017.01536)
Supplement: Supplementary file 6 [file Table6.DOC]

**Table S6. Percent of the ‘rare’ pangenes shared with at least one additional closely related species at various identity cut-offs.**

| **Organism** | **Rare pangenes** | **Percent of the shared ‘rares’** | | |
| --- | --- | --- | --- | --- |
|  |  | **55% identity** | **65% identity** | **75% identity** |
| *E. cloacae* | 5278 | 41.66 | 33.44 | 24.78 |
| *E. coli* | 7596 | 37.60 | 30.94 | 24.14 |
| *K. pneumoniae* | 3692 | 46.83 | 39.08 | 29.69 |
| *S. enterica* | 4893 | 50.85 | 43.59 | 34.99 |
