# Supplementary material for: Horizontally Acquired Genes Are Often Shared between Closely Related Bacterial Species
Source: Front Microbiol. 2017 Aug 25;8:1536. doi: 10.3389/fmicb.2017.01536 (PMC5575156; doi:10.3389/fmicb.2017.01536)
Supplement: Supplementary file 8 [file Table8.DOC]

**Table S8.** Conservation of the ‘rare’ pangenes outside the four analyzed species. Pangenes not found outside the four studied species are denoted as ‘non-conserved’. Sharing group denotes number of additional studied species ‘rare’ pangenes are shared with.

| **Organism** | **Sharing group** | **Number of the ‘rare’ pangenes** | **Non - conserved ‘rares’ (%)** | **Conserved ‘rares’** |
| --- | --- | --- | --- | --- |
| *E. cloacae* | Unique | 3079 | 44.49 | 55.51 |
|  | 1 | 990 | 13.03 | 86.97 |
|  | 2 | 663 | 3.47 | 96.53 |
|  | 3 | 546 | 0.37 | 99.63 |
| *E. coli* | Unique | 4740 | 59.07 | 40.93 |
|  | 1 | 1499 | 24.08 | 75.92 |
|  | 2 | 815 | 3.19 | 96.81 |
|  | 3 | 542 | 0.55 | 99.45 |
| *K. pneumoniae* | Unique | 1963 | 39.23 | 60.77 |
|  | 1 | 771 | 12.97 | 87.03 |
|  | 2 | 547 | 2.56 | 97.44 |
|  | 3 | 411 | 0.24 | 99.76 |
| *S. enterica* | Unique | 2405 | 56.96 | 43.04 |
|  | 1 | 1185 | 23.80 | 76.20 |
|  | 2 | 762 | 3.41 | 96.59 |
|  | 3 | 541 | 0.74 | 99.26 |
