# Supplementary material for: Horizontally Acquired Genes Are Often Shared between Closely Related Bacterial Species
Source: Front Microbiol. 2017 Aug 25;8:1536. doi: 10.3389/fmicb.2017.01536 (PMC5575156; doi:10.3389/fmicb.2017.01536)
Supplement: Supplementary file 9 [file Table9.DOC]

**Table S9**. Comparison of differences in the ENC’ values between ‘rare’ pangenes shared by various number of studied species using one-sided Mann-Whitney-Wilcoxon test. ‘Rares’ found only in one studied species (unique) were marked as shared with ‘0’ other species. Only pangenes with all genes in the pangene group having length of at least 100 codons and relative standard deviation of ENC’ values for individual genes <= 10% were analyzed (see Materials and Methods).

| **Organism** | **Compared sharing groups** | **# of pangenes in the group** | **U-test W** | ***P-value*** |
| --- | --- | --- | --- | --- |
| *E. cloacae* | 0-1 | 2959 - 983 | 1687500 | 2.345E-14 |
|  | 1-2 | 983 - 653 | 357190 | 5.387E-05 |
|  | 2-3 | 653 - 538 | 178170 | 3.351E-01 |
| *E. coli* | 0-1 | 4628 - 1479 | 4156500 | < 2.2E-16 |
|  | 1-2 | 1479 - 812 | 674640 | 4.874E-07 |
|  | 2-3 | 812 - 528 | 222110 | 1.316E-01 |
| *K. pneumoniae* | 0-1 | 1910 - 761 | 829400 | 5.799E-09 |
|  | 1-2 | 761 - 536 | 214940 | 4.901E-02 |
|  | 2-3 | 536 - 402 | 122160 | 2.229E-04 |
| *S. enterica* | 0-1 | 2370 - 1171 | 1673700 | < 2.2E-16 |
|  | 1-2 | 1171 - 757 | 511610 | 5.056E-09 |
|  | 2-3 | 757 - 538 | 210140 | 1.632E-01 |
