# Supplementary material for: Horizontally Acquired Genes Are Often Shared between Closely Related Bacterial Species
Source: Front Microbiol. 2017 Aug 25;8:1536. doi: 10.3389/fmicb.2017.01536 (PMC5575156; doi:10.3389/fmicb.2017.01536)
Supplement: Supplementary file 10 [file Table10.DOC]

**Table S10**. Comparison of differences in the FOP values between ‘rare’ pangenes shared by various number of studied species using one-sided Mann-Whitney-Wilcoxon test. ‘Rares’ found only in one studied species (unique) were marked as shared with ‘0’ other species. Only pangenes with relative standard deviation of FOP values for individual genes <= 10% were analyzed (see Materials and Methods).

| **Organism** | **Compared sharing groups** | **# of pangenes in the group** | **U-test W** | ***P-value*** |
| --- | --- | --- | --- | --- |
| *E. cloacae* | 0-1 | 3048 – 977 | 1153200 | < 2.2E-16 |
|  | 1-2 | 977 – 647 | 282960 | 1.737E-04 |
|  | 2-3 | 647 – 525 | 160760 | 5.764E-02 |
| *E. coli* | 0-1 | 4702 - 1488 | 2549700 | < 2.2E-16 |
|  | 1-2 | 1488 - 805 | 501340 | 5.641E-11 |
|  | 2-3 | 805 - 523 | 204290 | 1.812E-01 |
| *K. pneumoniae* | 0-1 | 1953 - 764 | 641510 | 6.485E-09 |
|  | 1-2 | 764 - 535 | 185710 | 2.519E-03 |
|  | 2-3 | 535 - 404 | 92677 | 9.171E-05 |
| *S. enterica* | 0-1 | 2393 - 1170 | 1011600 | < 2.2E-16 |
|  | 1-2 | 1170 - 746 | 357210 | 9.904E-12 |
|  | 2-3 | 746 - 528 | 181060 | 7.024E-03 |
