# Supplementary material for: Horizontally Acquired Genes Are Often Shared between Closely Related Bacterial Species
Source: Front Microbiol. 2017 Aug 25;8:1536. doi: 10.3389/fmicb.2017.01536 (PMC5575156; doi:10.3389/fmicb.2017.01536)
Supplement: Supplementary file 11 [file Table11.DOC]

**Table S11**. Percent of ‘rare’ pangenes in various sharing groups that could be assigned to COG functional categories using 70% identity cut-off or had functional annotation, besides “hypothetical protein”, in the NCBI database.

| **Organism** | **Sharing group** | **Number of ‘rares’ in the group** | **% of ‘rares’ assigned to any COG category** | **% of rares that have functional annotation** |
| --- | --- | --- | --- | --- |
| *E. cloacae* | Unique | 3079 | 15.10 | 33.00 |
|  | 1 | 990 | 33.54 | 56.97 |
|  | 2 | 663 | 41.33 | 64.86 |
|  | 3 | 546 | 45.05 | 68.68 |
| *E. coli* | Unique | 4740 | 9.96 | 37.81 |
|  | 1 | 1499 | 25.68 | 54.57 |
|  | 2 | 815 | 37.42 | 59.75 |
|  | 3 | 542 | 42.44 | 68.63 |
| *K. pneumoniae* | Unique | 1963 | 18.54 | 37.24 |
|  | 1 | 771 | 35.28 | 56.94 |
|  | 2 | 547 | 32.72 | 65.08 |
|  | 3 | 411 | 46.47 | 67.15 |
| *S. enterica* | Unique | 2405 | 7.82 | 32.64 |
|  | 1 | 1185 | 17.05 | 51.98 |
|  | 2 | 762 | 28.08 | 61.02 |
|  | 3 | 541 | 41.40 | 64.88 |
