# Supplementary material for: Horizontally Acquired Genes Are Often Shared between Closely Related Bacterial Species
Source: Front Microbiol. 2017 Aug 25;8:1536. doi: 10.3389/fmicb.2017.01536 (PMC5575156; doi:10.3389/fmicb.2017.01536)
Supplement: Supplementary file 12 [file Table12.DOC]

**Table S12**. Comparison of differences in the average length of ‘rare’ pangenes shared by various number of studied species using one-sided Mann-Whitney-Wilcoxon test. ‘Rares’ found only in one studied species (unique) were marked as shared with ‘0’ other species.

| **Organism** | **Compared sharing groups** | **# of pangenes in the group** | **U-test W** | ***P-value*** |
| --- | --- | --- | --- | --- |
| *E. cloacae* | 0-1 | 3079 - 990 | 1314400 | 3.476E-11 |
|  | 1-2 | 990 - 663 | 333760 | 7.210E-01 |
|  | 2-3 | 663 - 546 | 192590 | 9.725E-01 |
| *E. coli* | 0-1 | 4740 - 1499 | 3036200 | < 2.2E-16 |
|  | 1-2 | 1499 - 815 | 595750 | 1.627E-01 |
|  | 2-3 | 815 - 542 | 231830 | 9.396E-01 |
| *K. pneumoniae* | 0-1 | 1963 - 771 | 661010 | 1.275E-07 |
|  | 1-2 | 771 - 547 | 218790 | 8.777E-01 |
|  | 2-3 | 547 - 411 | 117120 | 8.668E-01 |
| *S. enterica* | 0-1 | 2405 - 1185 | 1299800 | 9.13E-06 |
|  | 1-2 | 1185 - 762 | 422240 | 7.857E-03 |
|  | 2-3 | 762 - 541 | 212230 | 8.193E-01 |
