# Supplementary material for: Horizontally Acquired Genes Are Often Shared between Closely Related Bacterial Species
Source: Front Microbiol. 2017 Aug 25;8:1536. doi: 10.3389/fmicb.2017.01536 (PMC5575156; doi:10.3389/fmicb.2017.01536)
Supplement: Supplementary file 13 [file Table13.DOC]

**Table S13.** Comparison of differences in the ENC’, FoP and average pangene length between unique ‘rare’ pangenes not found in any additional species within our database of fully sequenced bacterial genomes (‘ORFans’) and unique ‘rares’ found to be conserved outside of the four studied species (‘conserved’). The comparison was done using one-sided Mann-Whitney-Wilcoxon test.

| **Organism** | **Sharing group** | **ENC’** | | | **FOP** | | | **Average pangene length** | | |
| --- | --- | --- | --- | --- | --- | --- | --- | --- | --- | --- |
|  |  | **# of pangenes** | **U-test W** | ***P-value*** | **# of pangenes** | **U-test W** | ***P-value*** | **# of pangenes** | **U-test W** | ***P-value*** |
| *E. cloacae* | ORFans- Conserved | 1298 - 1661 | 1266400 | < 2.2E-16 | 1361 – 1687 | 871310 | < 2.2E-16 | 1370 - 1709 | 998860 | 1.207E-12 |
| *E. coli* | ORFans - Conserved | 2751 - 1877 | 3079200 | < 2.2E-16 | 2782 - 1920 | 2185300 | < 2.2E-16 | 2800 - 1940 | 2454100 | 7.888E-09 |
| *K. pneumoniae* | ORFans - Conserved | 741 - 1169 | 567080 | < 2.2E-16 | 766 - 1187 | 266050 | < 2.2E-16 | 770 - 1193 | 378130 | 1.796E-11 |
| *S. enterica* | ORFans - Conserved | 1351 - 1019 | 790530 | 2.885E-10 | 1363 - 1030 | 564260 | < 2.2E-16 | 1370 - 1035 | 660370 | 1.972E-03 |
